# The Attitudes of Chinese Cancer Patients and Family Caregivers toward Advance Directives

**DOI:** 10.3390/ijerph13080816

**Published:** 2016-08-11

**Authors:** Qiu Zhang, Chuanbo Xie, Shanghang Xie, Qing Liu

**Affiliations:** 1 Department of Health Service Management, Public Health School of Sun Yat-sen University, Guangzhou 510060, China; Qiu.z@yahoo.com; 2 State Key Laboratory of Oncology in South China, Department of Preventive Oncology, Sun Yat-sen University Cancer Center, Guangzhou 510060, China; xiechb@sysucc.org.cn (C.X.); Xieshh@sysucc.org.cn (S.X.)

**Keywords:** advance directives, Chinese, attitudes, cancer patients, family caregivers

## Abstract

Advance directives (ADs) have been legislated in many countries to protect patient autonomy regarding medical decisions at the end of life. China is facing a serious cancer burden and cancer patients’ quality at the end of life should be a concern. However, limited studies have been conducted locally to gather information about attitudes toward ADs. The purpose of this study was to investigate the attitudes of Chinese cancer patients and family caregivers toward ADs and to explore the predictors that are associated with attitudes. The study indicated that although there was low awareness of ADs, most cancer patients and family caregivers had positive attitudes toward ADs after related information was explained to them. Participants preferred to discuss ADs with medical staff when they were diagnosed with a life-threatening disease. Preferences for refusing life-sustaining treatment and choosing Hospice-Palliative Care (HPC) at the end of life would increase the likelihood of agreeing with ADs. This suggests that some effective interventions to help participants better understand end-of-life treatments are helpful in promoting ADs. Moreover, the development of HPC would contribute to Chinese cancer patients and family caregivers agreeing with ADs.

## 1. Introduction

Over the past six years, Chinese society has been discussing and debating the necessity of advance directives (ADs) in Mainland China. An advance directive is an umbrella term that includes living wills, durable power of attorney for health care, or combinations of both [1]. A living will is a written document to inform others what type of medical care patients wish to receive when they become terminally ill or permanently unconscious. A durable power of attorney for health care, also known as a health care proxy, is a document in which a patient appoints another person to make medical treatment decisions for him. ADs document patients’ preferences for end-of-life decisions, based on patient autonomy and self-determination. Many countries have already developed legislation for ADs, such as the United States [2], the United Kingdom [3], Germany [4], and particularly Singapore and Taiwan, which have traditional cultures similar to those of China. In Singapore, the Advance Medical Directives (AMD) Act was passed in 1996. This act established that anyone residing in Singapore who was at least 21 years of age and of sound of mind could sign an AMD [5]. In Taiwan, the Statute for Palliative Care was enacted in 2000 to provide patients and their family surrogates with the right to make do-not-resuscitate (DNR) decisions [6]. However, the development of ADs in mainland China has been very slow, and there is no case law regarding the validity of an advance directive.

The “World Cancer Report 2014”, published by the World Health Organization: (WHO), claimed that in 2012, nearly half of all new cancer cases worldwide occurred in Asia and most of those individuals lived in China [7]. The Annual report on the status of cancer in China (2010) also indicated that China was facing a serious cancer burden and prevention and control should be enhanced [8]. The absence of ADs may lead to unwanted aggressive treatment after a patient’s decision-making ability has been lost [9], and this unwanted aggressive care has been associated with poorer end-of-life quality and could [10] even influence the cost of terminal hospitalization [11]. Thus, ADs are an essential tool to record and respect patient’s preferences for end-of-life treatment.

At present, legal and ethical aspects are the focus of attention in discussions about ADs in mainland China. However, limited quantitative studies have been conducted locally to gather information on attitudes and perceptions toward ADs. Although some related studies have been conducted in Hong Kong, Taiwan and a few Chinese-American communities, it may not be suitable to apply their findings to the local context on the Chinese population in the wake of industrialization and aging.

The objects of this study were to assess the attitudes and perceptions toward ADs among cancer patients and family caregivers and to investigate the predictors that are associated with those attitudes.

## 2. Materials and Methods

### 2.1. Participants and Data Collection

Face-to-face interviews were conducted using a structured questionnaire. We recruited participants by convenience sampling from inpatients at seven organ-specific departments (i.e., stomach, lung, liver, colorectum, breast, uterus, and nasopharynx) in the Sun Yat-sen University Cancer Center (SYSUCC). SYSUCC is the largest integrated cancer center in the whole of Southern China for cancer care, education, research and prevention. Seven trained nurses from those departments were employed to give an information letter and a consent form to eligible cancer patients and family caregivers. In addition, they also conducted the interview and recorded participants’ responses after recruitment. Patients were identified by medical records, and family caregivers were identified by having consented to participate in this research. They were recruited separately. The inclusion criteria for cancer patient were: (1) age above 18 years old; (2) had a verified cancer diagnosis; (3) a level of alertness that permitted participation in an interview of 15–25 min; (4) ability to understand Chinese; and (5) providing written informed consent. Family caregivers, defined as a patient’s spouse, adult child, sibling, parent, and other relative, must be 18 years of age or older, be identified as a main caregiver who provided informal care to a cancer patient, and have consented to participate in this research. The exclusion criteria for participants were the presence of a known severe mental disorder and not being well enough to complete the questionnaire.

The interviews were conducted for cancer patients at the bedside, and for family caregivers in the lounge area. Data collection took place between February 2016 and March 2016. This study was approved by the ethics committee of Sun Yat-sen University Cancer Center (B2016-003-01) and performed in accordance with the Declaration of Helsinki. The purpose and procedure of the study were carefully explained before participants were interviewed. Informed consent was obtained via signature to ensure that each respondent would participate in the study on a voluntary basis. They were also assured of anonymity and were given the right to withdraw from the interview whenever necessary.

### 2.2. Measurements

We developed a structured questionnaire to explore the attitudes of cancer patients and family caregivers toward advance directives. The questionnaire was modified from the measurement instrument created by Akabayashi et al. [12]. Two items about organ donations were deleted and three items about family functioning and Hospice-Palliative Care were added based on the consideration of Chinese traditional culture and poor prognosis in cancer patients. Ultimately, 20 items were used to measure the participants’ attitudes regarding ADs. The questionnaire’s reliability and content validity were established through a pilot test-retest and by an expert panel before the survey was conducted. The panel members were experts in end-of-life care and ADs and represented the disciplines of nursing, law and bioethics.

The questionnaire was divided into three categories: (1) socio-demographic characteristics (11 items): (sex, age, marital status, annual income, education level, religiousness, living area, living arrangement, family functioning, duration of cancer and awareness of ADs); (2) attitudes of the participants toward ADs (one item), and perceptions of ADs (four items): the optimal timing of ADs, degree of leeway regarding patients’ ADs, the optimal role to communicate about ADs and attitudes toward legalization; (3) opinions on end-of-life decisions (four items), such as disclosure of terminal illness, life-sustaining treatments and Hospice-Palliative Care (HPC).

The APGAR questionnaire assesses family functioning in five dimensions: Adaptation, Partnership, Growth, Affection, and Resolve. The Chinese version of the Family APGAR Index (C-APGAR) is based on the original version developed by Smilkstein [13]. Each item is scored on a three-point scale (0 = hardly ever, 1 = sometimes, 2 = almost always). The sum of the five is the total score (range = 0–10). High scores indicate good family support. It has been found to be a reliable tool when used with cancer patients, with an alpha coefficient of 0.88 reported in a study of bone marrow transplant survivors [14], and was used to measure the family function as one of the risk factors associated with depression in a large-scale representative Taiwanese adolescent population [15], its Cronbach’s alpha was 0.843 and two-week test-retest reliability was 0.724. The internal consistency coefficient was 0.69 in the present study.

To explore participants’ preferences regarding Hospice-Palliative Care (HPC), we adopted a conceptual framework from previous studies [16,17,18]. In recruitment, each of participants was provided a booklet for describing the concept and goal of HPC. In addition, our interviewers would give detailed explanations to participants if they had any question regarding HPC. Participants were asked about their opinions regarding HPC in two sentences which followed the guidelines of American Academy of Hospice and Palliative Medicine (AAHPM) [19] and was reviewed by the expert panel: (1) HPC could provide relief from pain and other distressing symptoms, and improve the quality of life of terminal cancer patients; and (2) HPC is a better choice than aggressive treatment for terminal cancer patients. The response format used a three-point Likert scale with “agree”, “no preference”, and “disagree”. The two items’ consistency coefficient was 0.76. The similar statements were used to examine the American community-dwelling older adults’ desire for hospice care and beliefs about the type of care hospice provides, and its Cronbach’s alpha was 0.74 [18]. Those were also used with an alpha coefficient of 0.87, to solicit professionals’ perceptions of the barriers to HPC, and to relate their perceptions about advance directives to their perceived obstacles to HPC [17]. Opinions about life-sustaining treatment were measured by making a choice when given the following situation: if you were diagnosed as having an incurable and irreversible illness, disease, or condition, what would be your choice about life-sustaining treatment in the dying stage? The possible answers included: request, refuse and leave to other people to decide. The items’ consistency coefficient was 0.73 in this study.

A total of 20 questions were asked to obtain participants’ attitudes regarding ADs. Most of questionnaire items were put to cancer patients and family caregivers similarly.

### 2.3. Statistical Analysis

Descriptive statistics were used to calculate the means and the standard deviations of the continuous variables and percentages and frequencies of the categorical variables. In order to examine the differences of the proportion in participants’ attitudes and perceptions toward ADs, the Chi-square test was applied. Bivariate analyses were used to test associations with attitudes toward ADs. To find out which of the socio-demographic characteristics and preferences variables can be used as independent predictors for the attitudes toward ADs, We next performed a multivariate analyses. Odds ratios (ORs) and their associated 95% confidence intervals (CIs) were calculated accordingly.

All *p*-values less than 0.05 for two-tailed test were regarded as statistically significant. The statistical software SPSS for Windows version 20.0 (SPSS Inc., Chicago, IL, USA) was used for all data analysis.

## 3. Results

### 3.1. Characteristics of the Study Subjects

We interviewed a total of 424 participants, including 209 cancer patients and 215 family caregivers. The participants’ characteristics are presented in Table 1. Most of the participants were married and lived with their families. Seventy-five percent of participants were nonreligious, and only a minority (9%) had annual income over US $13,000.

### 3.2. Attitudes and Perceptions toward Advance Directives

Participants’ responses to these questions are shown in Table 2. Most participants (74%) agreed with advance directives (ADs). More than 80% answered that “when they were diagnosed with a life-threatening disease” was the optimal time for completing ADs, and less than 10% answered “when healthy”. An overwhelming majority (>85%) thought that the optimal role to communicate about ADs with was a medical staff member. Additionally, 72% of participants claimed that if they had created an AD, they would like to be treated according to their ADs “as much as possible”; only 6% responded with “in absolute accordance”, and 22% indicated “just as a reference”. Furthermore, 71% of participants agreed with the legalization of ADs. Regarding the items described above, there were no statistically significant differences among cancer patients and family caregivers.

### 3.3. Factors Associated with the Participants’ Attitudes toward ADs

Not living with family was a characteristic more predominantly found among those with positive attitudes toward ADs than those with negative attitudes. No differences in gender, age group, education level or other characteristics were observed between the two groups with different attitudes (Table 3). In addition, except for the opinion on whether HPC can provide relief from pain and improve the quality of life for terminal cancer patients, the distributions of other attitude variables were different between the group that agreed with ADs and the group that disagreed with ADs, as shown in Table 4.

### 3.4. Predictors of the Participants’ Attitudes towards ADs

Univariate and multivariate analyses to predict attitudes of participants toward ADs are presented in Table 5. Data analysis showed that not living with family, longer duration of cancer, agreeing with terminal illness disclosure, having heard of ADs, preferring life-sustaining treatment, agreeing that HPC is a better choice than aggressive treatment for terminal cancer patients, and lower family APGAR scores were independent variables that predicated the positive attitudes of participants. For example, participants who had heard of ADs before were 5.94 times more likely to agree with ADs compared to those who had not heard of them (adjusted odds ratio (AOR): 5.94; 95% CI: 1.83, 19.27), and participants who lived with family were 72% less likely to agree with ADs compared to those not living with family (AOR: 0.27; 95% CI: 0.12, 0.59). Similarly, compared with participants who preferred to request life-sustaining treatment, those who refused were 38.21 times more likely to agree with ADs (AOR: 38.21; 95% CI: 11.82, 123.54).

## 4. Discussion

This study provides valuable insights into the attitudes and perceptions of Chinese cancer patients and family caregivers toward ADs, especially given the limited research in mainland China. In the study, most of the participants (86.1%) had never heard of ADs before this survey, but after being informed of the concept, almost three-quarters of them showed positive attitudes toward ADs. The results are similar to those of another study, which found 92.7% of Korean patients and family caregivers agreed with ADs and only 16.2% of them heard of the terms [20]. Chu L et al. also found that after explaining the concept of ADs, 88.0% of Chinese nursing home residents in Hong Kong agreed that it would be good to have an advance directive to express their preferences for end-of-life care decisions [21]. These findings are somewhat inconsistent with a previous study showing that non-western racial groups were less likely than western populations to support ADs [22,23]. China’s modernization during the past three decades (1978–2008) has had a great impact on the changes in Chinese behaviors and values. It appears that an individualistic self-determination has been on the rise in China [24]. Thus, there is a growing concern about respect for patient autonomy and self-determination. Additionally, the lack of awareness about ADs in this study also suggests the need for enhancing public education programs. Various means of promoting ADs should also be a topic of further investigation.

Our study revealed that many participants (71.0%) agreed with legislation regarding ADs. This proportion was much higher than the 49% found in another study about Hong Kong Chinese elders [25], but is consistent with a previous study by Ivo and his partners, where 79.3% of cancer patients acknowledged the need for a legally authorized “ADs” for medical decision-making if they became unconscious or could not communicate [26]. Although there is no law regarding ADs in China, the participants showed openness to ADs after the concept was explained to them. Furthermore, our data showed that most participants (87.6%) emphasized the binding force of an AD, but only 5.9% responded with “in absolute accordance”. The proportion is lower than 39% in the study by Sehgal et al. [27] and 13.2% in another study by Akabayashi et al. [12]. Because an AD is not a mandatory document in China, it is not surprising that the percentage of people who wish their ADs to be followed strictly is relatively lower.

In the study, participants tended to regard diagnosis with a life-threatening disease as an optimal time for completing ADs, perhaps because people who describe their health as poor or who become ill are more likely to think about their end-of-life treatments and consider completing ADs. Although other studies have found that a family member was the most common person to communicate with about ADs in patients in countries with East Asian cultures [28,29], this study indicated that participants tended to regard medical staff as the optimal role to communicate with about ADs. This conclusion also supports the findings of previous studies [30,31,32]. Van Oorschot et al. [32] reported that nearly half of cancer patients considered ADs as a door for better patient-physician communication. Pollack et al. [30] demonstrated that most Maryland adults had a preference for end-of-life care and wanted to discuss these issues with their physicians. Sahm et al. [31] found that oncologists should initiate a discussion about an AD when the course of the illness seems to make this appropriate. Hence, patients and family caregivers usually expect that their physicians should take the initiative in addressing the topic of ADs [33,34], and when medical staff have communicated with patients, they have been more likely to complete ADs [35,36].

Multivariate analysis revealed approximately seven factors associated with participants’ attitudes toward ADs. The strongest predictor for agreeing with ADs was refusing life-sustaining treatment at the end of life; this finding is consistent with previous studies [12,20,25]. Those who do not prefer life-sustaining treatment may be concerned that, if they do not express their wishes via an AD, it is possible that the family would choose life-sustaining treatment for them out of love. In this regard, providing sufficient information about life-sustaining treatment to patients and their family members is important to help patients assess their end-of-life preferences [37]. Two other independent predictors included having heard of ADs and preferring disclosure of terminal illness, which suggests that when people have sufficient knowledge about the possible options for end-of-life treatment, they can make good decisions based on their wishes [38]. In addition, a duration of cancer of more than three years would cause cancer patients and family caregivers to pay more attention to end-of-life treatment and to have more chances to communicate about this issue, which could contribute to the completion of ADs [32]. Living with family and higher family APGAR scores were risk predictors for positive attitudes toward ADs. This indicates that individuals with better-functioning family were less likely to prefer ADs, which is consistent with previous studies [20,39]. This is because the family is the smallest unit of identity in Chinese traditional culture, and patients often choose their children or spouse as a proxy to make decisions for them [6,26]. Therefore, although it appears that emerging sociological changes in family dynamics have increased individualism in patients’ autonomy [40], family still plays an important role in decisions at a patient’s end of life in China. The last predictor was supporting HPC as a better choice than aggressive treatment for terminal cancer patients. Several studies showed that many patients with terminal illnesses preferred to choose palliative care to relieve symptoms, improve their quality of life, and strive for a peaceful death [41,42]. In this regard, patients need ADs to refuse aggressive treatment and instead choose HPC to make themselves comfortable at the end of their life.

## 5. Conclusions

We found that although there was low awareness of ADs, most cancer patients and family caregivers had positive attitudes toward ADs after related information was explained to them. This suggests that some effective interventions to help participants understand end-of-life treatments better are helpful in promoting ADs in China. Participants in this study preferred to discuss end-of-life treatment with medical staff when they became worse. In addition, this study revealed there was a strong need for legal measures in setting up an AD. The results were also able to delineate seven factors significantly associated with positive attitudes. Preferences for refusing life-sustaining treatment and choosing HPC at end of life would increase the likelihood of agreeing with ADs. Therefore, the promotion of ADs among cancer patients in China should act in concert with the development of HPC.

There are several limitations of this study, and, therefore, the findings should be interpreted with caution. First, we used a convenience sample of participants in a single cancer center. Thus, their views may not represent the general population of cancer patients and family caregivers in China. Second, the findings are susceptible to non-respondent bias because data were collected from respondents who agreed to participate. Third, although we used a questionnaire modified from previous studies and tested its reliability and validity carefully before conducting the interview, some measures’ reliabilities such as the Family APGAR were low in our study. In addition, since APGAR is only a screening tool, the assessment of family functioning might therefore be subject to measurement errors. Next we will make some modifications and refine this survey instrument, and then conduct additional research with a large national sample of cancer patients and family caregivers in China to verify the current findings further.

## Figures and Tables

**Table 1:** Characteristics of the study subjects.

Characteristics	Total (*n* = 424)	Patients (*n* = 209)	Family Caregivers (*n* = 215)
Gender, *n* (%)			
Male	229 (54.0)	111 (53.1)	118 (54.9)
Female	195 (46.0)	98 (46.9)	97 (45.1)
Age group, *n* (%)			
<45 years	198 (46.7)	43 (20.6)	155 (72.1)
≥45 years	226 (53.3)	166 (79.4)	60 (27.9)
Education level, *n* (%)			
Junior school or less	184 (43.4)	113 (54.1)	71 (33.1)
Senior school	143 (33.7)	66 (31.6)	77 (35.8)
College or higher	97 (22.9)	30 (14.3)	67 (31.2)
Annual income in US $, *n* (%)			
<3500	193 (45.5)	96 (45.9)	97 (45.1)
3500–13,000	192 (45.3)	99 (47.4)	93 (43.3)
>13,000	39 (9.2)	14 (6.7)	25 (11.6)
Marital Status, *n* (%)			
Married	326 (76.9)	165 (78.9)	161 (74.9)
Single, Bereaved, Divorced	98 (23.1)	44 (21.1)	54 (25.1)
Religiousness, *n* (%)			
Religious	106 (25.0)	52 (24.9)	54 (25.1)
Nonreligious	318 (75.0)	157 (75.1)	161 (74.9)
Living area, *n* (%)			
Urban	107 (25.2)	65 (31.1)	42 (19.5)
Suburban	196 (46.2)	78 (37.3)	118 (54.9)
Rural	121 (78.5)	66 (31.6)	55 (25.6)
Living with family, *n* (%)			
Yes	323 (76.2)	181 (86.6)	142 (66.0)
No	101 (23.8)	28 (13.4)	73 (34.0)
Duration of cancer ^a^, *n* (%)			
<3 years	252 (59.43)	174 (83.25)	78 (36.3)
≥3 years	172 (40.57)	35 (16.75)	137 (63.7)
Awareness of ADs ^b^, *n* (%)			
Unaware	365 (86.08)	183 (87.56)	182 (84.6)
Aware	59 (13.92)	26 (12.44)	33 (15.4)
Family APGAR, Mean ± SD ^c^	7.69 ± 1.05	7.29 ± 1.04	8.07 ± 0.91

^a^ For patients, “Duration of cancer” means time since their initial cancer diagnosis. For family caregiver, “Duration of cancer” means time since their family member’s initial cancer diagnosis rather than themselves; ^b^ ADs = advance directives; ^c^ SD = standard deviation.

**Table 2:** Participants’ attitudes and perceptions toward ADs.

Variables	Total (*n* = 424)	Patients (*n* = 209)	Family Caregivers (*n* = 215)	*p*
Attitudes toward ADs, *n* (%)				0.549
Disagree	111 (26.2)	52 (24.9)	59 (27.4)	
Agree	313 (73.8)	157 (75.1)	156 (72.6)	
The optimal timing of ADs, *n* (%)				0.665
No idea	25 (5.9)	12 (5.7)	13 (6.4)	
When diagnosed with a life-threatening disease	357 (84.2)	179 (85.7)	178 (82.8)	
When healthy	42 (9.9)	18 (8.6)	24 (11.2)	
Degree of leeway regarding patients’ ADs, *n* (%)				0.085
In absolute accordance	25 (5.9)	15 (7.2)	10 (4.7)	
As much as possible	304 (71.7)	156 (74.6)	148 (68.8)	
Just as a reference	95 (22.4)	38 (18.2)	57 (26.5)	
The optimal role to communicate about ADs, *n* (%)				0.084
The health care proxy	5 (1.2)	5 (2.4)	0 (0)	
Family members or relatives	55 (13.0)	27 (12.9)	28 (13.0)	
Medical staff	364 (85.8)	177 (84.6)	187 (87.0)	
Attitudes toward legalization, *n* (%)				0.893
Disagree	123 (29.0)	60 (28.7)	63 (29.3)	
Agree	301 (71.0)	149 (71.3)	152 (70.7)	

**Table 3:** Characteristics associated with participants’ attitudes toward ADs.

Variables	Attitudes		*p*
	Agree with ADs (*n* = 313)	Disagree with ADs (*n* = 111)	
Group, *n* (%)			
Patients	157 (50.2)	52 (46.8)	
Family caregivers	156 (49.8)	59 (53.2)	0.549
Gender, *n* (%)			
Male	168 (53.7)	61 (55.0)	
Female	145 (46.3)	50 (45.0)	0.816
Age group, *n* (%)			
<45 years	139 (44.4)	59 (53.2)	
≥45 years	174 (55.6)	52 (46.8)	0.113
Education level, *n* (%)			
Junior school or less	133 (42.5)	51 (45.9)	
Senior school	107 (34.2)	36 (32.4)	
College or higher	73 (23.3)	24 (21.6)	0.817
Annual income in US $, *n* (%)			
<3500	136 (43.5)	57 (51.4)	
3500–13,000	149 (47.6)	43 (38.7)	
>13,000	28 (8.9)	11 (9.9)	0.269
Marital Status, *n* (%)			
Single, Bereaved, Divorced	77 (24.6)	21 (18.9)	
Married	236 (75.4)	90 (81.1)	0.222
Religiousness, *n* (%)			
Nonreligious	228 (72.8)	90 (81.1)	
Religious	85 (27.2)	21 (18.9)	0.085
Living area, *n* (%)			
Urban	85 (27.2)	22 (19.8)	
Suburban	144 (46.0)	52 (46.8)	
Rural	84 (26.8)	37 (33.3)	0.226
Living with family, *n* (%)			
No	85 (27.2)	16 (14.4)	
Yes	228 (72.8)	95 (85.6)	0.007
Duration of cancer, *n* (%)			
<3 years	175 (55.9)	77 (69.4)	
≥3 years	138 (44.1)	34 (30.6)	0.013
Awareness of ADs, *n* (%)			
Unaware	259 (82.7)	106 (95.5)	
Aware	54 (17.3)	5 (4.5)	0.001
Family APGAR, Mean ± SD	7.52 (1.01)	8.16 (1.01)	<0.001

**Table 4:** Opinions on end-of-life decisions associated with participants’ attitudes toward ADs.

Variables	Attitudes		*p*
	Agree with ADs ( *n* = 313)	Disagree with ADs ( *n* = 111)	
Disclosure of terminal illness ^a^			
Disagree	241 (77.0)	106 (95.5)	
Agree	72 (23.0)	5 (4.5)	<0.001
Opinion on whether HPC ^b^ can provide relief from pain and improve the quality of life for terminal cancer patients			
No preference	21 (6.7)	14 (12.6)	
Agree	292 (93.3)	97 (87.4)	0.052
Opinion on whether HPC is a better choice for terminal cancer patients			
No preference	58 (18.5)	35 (31.5)	
Agree	255 (81.5)	76 (68.5)	0.004
Opinions on whether use life-sustaining ^c^ treatment at patients’ end of life			
Request	21 (6.7)	36 (32.4)	
Refuse	122 (39.0)	6 (5.4)	
Leave to others ^d^ to decide	170 (54.3)	69 (62.2)	<0.001

^a^ “Disclosure of terminal illness” is defined as truth-telling to incurable cancer patients with diagnosis, treatment and prognosis; ^b^ HPC = hospice-palliative care; ^c^ Opinions about life-sustaining treatment” is described as: If you were diagnosed as having an incurable and irreversible illness, disease, or condition, what would be your choice about life-sustaining treatment in the dying stage? ^d^ “Other people” here was primarily described as a participant’s spouse, adult child, sibling, parent, or other relative.

**Table 5:** Logistic regression analysis of independent predictors of attitudes toward ADs (agree vs. disagree).

Variables	Univariate Analysis		Multivariate Analysis	
	OR (95% CI)	*p*	AOR (95% CI)	*p*
Living with family (yes vs. no)	0.45 (0.25, 0.81)	0.007	0.27 (0.12, 0.59)	0.001
Duration of cancer, years (≥3 years vs. <3 years)	1.79 (1.13, 2.83)	0.013	2.06 (1.10, 3.83)	0.023
Disclosure of terminal illness (yes vs. no)	6.33 (2.49, 16.13)	<0.001	5.72 (1.98, 16.56)	0.001
Family APGAR ^a^	0.50 (0.39, 0.65)	<0.001	0.65 (0.48, 0.89)	0.006
Awareness of ADs (aware vs. unaware)	4.42 (1.72, 11.37)	0.001	5.94 (1.83, 19.27)	0.003
Opinions on whether use life-sustaining treatment at patients’ end of life				
Request	1.00		1.00	
Refuse	34.86 (13.08, 92.92)	<0.001	38.21 (11.82, 123.54)	<0.001
Leave to others to decide	4.22 (2.30, 7.75)	<0.001	4.37 (2.06, 9.26)	<0.001
Opinions on whether HPC is a better choice for terminal cancer patients (agree vs. no preference)	2.03 (1.24, 3.31)	0.004	2.68 (1.31, 5.46)	0.007

^a^ Family APGAR includes five dimensions, each of them is scored on a three-point scale (0 = hardly ever, 1 = sometimes, 2 = almost always). The sum of the five is the total score (range = 0–10); ROR = odds ratio; AOR = adjusted odds ratio; CI = confidence intervals.

## References

[1] Clements J.M. (2009). Patient perceptions on the use of advance directives and life prolonging technology. Am. J. Hosp. Palliat. Med..

[2] Brown B.A. (2003). The history of advance directives a literature review. J. Gerontol. Nurs..

[3] Affairs D.F.C. (2007). Mental Capacity Act 2005 Code of Practice.

[4] Wiesing U., Jox R., Hessler H., Borasio G. (2010). A new law on advance directives in Germany. J. Med. Ethics.

[5] Tay M., Chia S.E., Sng J. (2010). Knowledge, attitudes and practices of the advance medical directive in a residential estate in Singapore. Ann. Acad. Med. Singap..

[6] Lo Y., Wang J., Liu L., Wang C. (2010). Prevalence and related factors of do-not-resuscitate directives among nursing home residents in Taiwan. J. Am. Med. Dir. Assoc..

[7] Stewart B., Wild C.P. (2015). World Cancer Report 2014.

[8] Chen W., Zheng R., Zhang S., Zhao P., Zeng H., Zou X., He J. (2014). Annual report on status of cancer in China, 2010. Chin. J. Cancer Res..

[9] Matsuyama R., Reddy S., Smith T.J. (2006). Why do patients choose chemotherapy near the end of life? A review of the perspective of those facing death from cancer. J. Clin. Oncol..

[10] Teno J.M., Gruneir A., Schwartz Z., Nanda A., Wetle T. (2007). Association between advance directives and quality of end-of-life care: A national study. J. Am. Geriatr. Soc..

[11] Weeks W.B., Kofoed L.L., Wallace A.E., Welch H.G. (1994). Advance directives and the cost of terminal hospitalization. Arch. Intern. Med..

[12] Akabayashi A., Slingsby B., Kai I. (2003). Perspectives on advance directives in Japanese society: A population-based questionnaire survey. BMC Med. Ethics.

[13] Smilkstein G. (1978). The Family APGAR: A proposal for family function test and its use by physicians. J. Fam. Pract..

[14] Chang G., McGarigle C., Spitzer T.R., McAfee S.L., Harris F., Piercy K., Goetz M.A., Antin J.H. (1998). A comparison of related and unrelated marrow donors. Psychosom. Med..

[15] Lin H.C., Tang T.C., Yen J.Y., Ko C.H., Huang C.F., Liu S.C., Yen C.F. (2008). Depression and its association with self-esteem, family, peer and school factors in a population of 9586 adolescents in southern Taiwan. Psychiatry Clin. Neurosci..

[16] Weisbord S.D., Carmody S.S., Bruns F.J., Rotondi A.J., Cohen L.M., Zeidel M.L., Arnold R.M. (2003). Symptom burden, quality of life, advance care planning and the potential value of palliative care in severely ill haemodialysis patients. Nephrol. Dial. Transplant..

[17] Feeg V.D., Elebiary H. (2005). Exploratory study on end-of-life issues: Barriers to palliative care and advance directives. Am. J. Hosp. Palliat. Med..

[18] Johnson K.S., Kuchibhatla M., Tulsky J.A. (2008). What explains racial differences in the use of advance directives and attitudes toward hospice care?. J. Am. Geriatr. Soc..

[19] Holman G.H., Forman W.B. (2001). On the 10th anniversary of the organization of the American Academy of Hospice and Palliative Medicine (AAHPM): The first 10 years. Am. J. Hosp. Palliat. Med..

[20] Kim S.H. (2011). Factors influencing preferences of Korean people toward advance directives. Nurs. Ethics.

[21] Chu L., Luk J.K., Hui E., Chiu P.K., Chan C.S., Kwan F., Kwok T., Lee D., Woo J. (2011). Advance directive and end-of-life care preferences among Chinese nursing home residents in Hong Kong. J. Am. Med. Dir. Assoc..

[22] Kiely D.K., Mitchell S.L., Marlow A., Murphy K.M., Morris J.N. (2001). Racial and state differences in the designation of advance directives in nursing home residents. J. Am. Geriatr. Soc..

[23] Kwak J., Haley W.E. (2005). Current research findings on end-of-life decision making among racially or ethnically diverse groups. Gerontologist.

[24] Zhang Y.B., Lin M., Nonaka A., Beom K. (2005). Harmony, hierarchy and conservatism: A cross-cultural comparison of Confucian values in China, Korea, Japan, and Taiwan. Commun. Res. Rep..

[25] Ting F.H., Mok E. (2011). Advance directives and life-sustaining treatment: Attitudes of Hong Kong Chinese elders with chronic. Hong Kong Med. J..

[26] Ivo K., Younsuck K., Ho Y.Y., Sang-Yeon S., Seog H.D., Hyunah B., Kenji H., Xiaomei Z. (2012). A survey of the perspectives of patients who are seriously ill regarding end-of-life decisions in some medical institutions of Korea, China and Japan. J. Med. Ethics..

[27] Sehgal A., Galbraith A., Chesney M., Schoenfeld P., Charles G., Lo B. (1992). How strictly do dialysis patients want their advance directives followed?. JAMA.

[28] Ni P., Zhou J., Wang Z.X., Nie R., Phillips J., Mao J. (2014). Advance directive and end-of-life care preferences among nursing home residents in Wuhan, China: A cross-sectional study. J. Am. Med. Dir. Assoc..

[29] Malhotra C., Chan A., Do Y.K., Malhotra R., Goh C. (2012). Good end-of-life care: Perspectives of middle-aged and older Singaporeans. J. Pain Symptom Manag..

[30] Pollack K.M., Morhaim D., Williams M.A. (2010). The public’s perspectives on advance directives: Implications for state legislative and regulatory policy. Health Policy.

[31] Sahm S., Will R., Hommel G. (2005). What are cancer patients’ preferences about treatment at the end of life, and who should start talking about it? A comparison with healthy people and medical staff. Support. Care Cancer.

[32] Van Oorschot B., Schuler M., Simon A., Flentje M. (2012). Advance directives: Prevalence and attitudes of cancer patients receiving radiotherapy. Support. Care Cancer.

[33] Barnes K.A., Barlow C.A., Harrington J., Ornadel K., Tookman A., King M., Jones L. (2011). Advance care planning discussions in advanced cancer: Analysis of dialogues between patients and care planning mediators. Palliat. Support. Care.

[34] Rurup M.L., Onwuteaka-Philipsen B.D., van der Heide A., van der Wal G., Deeg D.J. (2006). Frequency and determinants of advance directives concerning end-of-life care in the Netherlands. Soc. Sci. Med..

[35] Wissow L.S., Belote A., Kramer W., Compton Phillips A., Kritzler R., Weiner J.P. (2004). Promoting advance directives among elderly primary care patients. J. Gen. Intern. Med..

[36] Johnston S.C., Pfeifer M.P., McNutt R., Adelman H.M., Wallach P.M., Boero J.F., Crnkovich D., Halbritter K.A., Johnston S.C., Layson R. (1995). The discussion about advance directives: Patient and physician opinions regarding when and how it should be conducted. Arch. Intern. Med..

[37] Kizawa Y., Tsuneto S., Hamano J., Nagaoka H., Maeno T., Shima Y. (2013). Advance directives and Do-Not-Resuscitate orders among patients with terminal cancer in palliative care units in japan a nationwide survey. Am. J. Hosp. Palliat. Med..

[38] High D.M. (1993). Why are elderly people not using advance directives?. J. Aging Health.

[39] Liu J.M., Lin W.C., Chen Y.M., Wu H.W., Yao N.S., Chen L.T., Whang-Peng J. (1999). The status of the do-not-resuscitate order in Chinese clinical trial patients in a cancer centre. J. Med. Ethics.

[40] Ho Z.J.M., Krishna L.K.R., Yee C.P.A. (2010). Chinese familial tradition and Western influence: A case study in Singapore on decision making at the end of life. J. Pain Symptom Manag..

[41] Curtis J.R. (2008). Palliative and end-of-life care for patients with severe COPD. Eur. Respir. J..

[42] Manfredi P.L., Morrison R.S., Morris J., Goldhirsch S.L., Carter J.M., Meier D.E. (2000). Palliative care consultations: How do they impact the care of hospitalized patients?. J. Pain Symptom Manag..

